# Diversification trajectories and paleobiogeography of Neogene chondrichthyans from Europe

**DOI:** 10.1017/pab.2022.40

**Published:** 2023-02-08

**Authors:** Jaime A. Villafaña, Marcelo M. Rivadeneira, Catalina Pimiento, Jürgen Kriwet

**Affiliations:** Vienna Doctoral School of Ecology and Evolution, Vienna, Austria; Centro de Investigación en Recursos Naturales y Sustentabilidad, Universidad Bernardo O’Higgins, Avenida Viel 1497, 8370993 Santiago, Chile; Laboratorio de Paleobiología, Centro de Estudios Avanzados en Zonas Áridas (CEAZA), Avenida Bernardo Ossandón 877, 1781681, Coquimbo, Chile; Departamento de Biología Marina, Facultad de Ciencias del Mar, Universidad Católica del Norte, Larrondo 1281, Coquimbo, Chile; Departamento de Biología, Universidad de La Serena, Avenida Raul Bitrán 1305, La Serena, Chile; Paleontological Institute and Museum, University of Zurich, CH-8006 Zurich, Switzerland; Department of Biosciences, Swansea University, Swansea SA28PP, United Kingdom; Smithsonian Tropical Research Institute, Balboa, Panama; Department of Palaeontology, University of Vienna, Althanstraβe 14, Geocenter, 1090 Vienna, Austria

## Abstract

Despite the rich fossil record of Neogene chondrichthyans (chimaeras, sharks, rays, and skates) from Europe, little is known about the macroevolutionary processes that generated their current diversity and geographical distribution. We compiled 4368 Neogene occurrences comprising 102 genera, 41 families, and 12 orders from four European regions (Atlantic, Mediterranean, North Sea, and Paratethys) and evaluated their diversification trajectories and paleobiogeographic patterns. In all regions analyzed, we found that generic richness increased during the early Miocene, then decreased sharply during the middle Miocene in the Paratethys, and moderately during the late Miocene and Pliocene in the Mediterranean and North Seas. Origination rates display the most significant pulses in the early Miocene in all regions. Extinction rate pulses varied across regions, with the Paratethys displaying the most significant pulses during the late Miocene and the Mediterranean and North Seas during the late Miocene and early Pliocene. Overall, up to 27% and 56% of the European Neogene genera are now globally and regionally extinct, respectively. The observed pulses of origination and extinction in the different regions coincide with warming and cooling events that occurred during the Neogene globally and regionally. Our study reveals complex diversity dynamics of Neogene chondrichthyans from Europe and their distinct biogeographic composition despite the multiple marine passages that connected the different marine regions during this time.

## Introduction

The formation of the Mediterranean Sea was the result of intense geographic and oceanographic changes that took place over the last 150 Myr (Rögl 1999). During the Mesozoic, the Tethys Ocean separated the continents of Laurasia and Gondwana until the end of the Eocene, when continental drift and Alpine orogeny caused it to vanish (Rögl 1999; Berra and Angiolini 2014). By the Oligocene, Europe’s marine areas reorganized into three distinct biogeographic provinces: Paratethys, Mediterranean, and Atlantic boreal (Steininger and Wessely 2000). The Paratethys formed a separate branch of the former Tethys Ocean in the north, and a proto–Mediterranean Sea formed in the south (Rögl 1998). As a consequence of these tectonic changes, Europe was largely covered by parts of the eastern Atlantic Ocean, the Mediterranean Sea, the Paratethys, and the North Sea, which together formed shallow epicontinental seas during the Neogene (23 to 2.6 Ma; Rögl 1998).

The connectivity of Europe’s main marine regions resulted in major environmental and biotic changes during the Neogene. The Mediterranean and Paratethys Seas experienced a series of connections and disconnections through sea passages from the Miocene to Pliocene (Rögl 1999). During the Burdigalian (20.43–15.97 Ma), the Paratethys was connected to the Atlantic Ocean, the North Sea, and the Mediterranean Sea, enabling faunal exchanges (Kroh 2007; Reinecke et al. 2011). At the end of the Burdigalian, the western part of the Paratethys fell dry, but the western and central Paratethys remained under marine conditions and connected to the Mediterranean Sea (Rögl 1999). From the Serravalian (13.82–11.63 Ma) onward, the Paratethys became gradually isolated from the Mediterranean Sea, and at the end of the Serravalian, the uplift of the Carpathian Mountains separated them (Harzhauser and Kowalke 2002; Harzhauser and Piller 2007). A gradual decrease of temperature and salinity caused a continuous development of endemic faunas in the Central Paratethys (Kroh 2007), and the closure of sea passages triggered the final isolation of the Paratethys and its transition from a marine to a freshwater environment. The Mediterranean Sea was affected by a gradual decrease of temperature and desiccation events during the Tortonian and Messinian (11.63–5.33 Ma; Rögl 1999), although marine organisms persisted throughout the three stages of the Messinian salinity crisis (e.g., Carnevale et al. 2019). The temperature drop continued through the Pliocene, reaching its lowest levels during the beginning of the Pleistocene glaciations (Westerhold et al. 2020). The waxing and waning of large high-latitude ice sheets caused intense fluctuations of sea levels during the Quaternary (Lambeck and Chappell 2001), including in the Mediterranean Sea (Brunović et al. 2020). As a result, marine faunas in Europe faced large and persistent environmental changes throughout the Neogene, which likely influenced their diversity and distribution.

Indeed, previous studies have regarded the intense climatic and oceanographic events of the European Neogene as the cause for the faunistic changes that occurred in that region during this time (Harzhauser et al. 2003; Moissette et al. 2006; Harzhauser and Piller 2007; Kroh 2007; Piller et al. 2007; Borgh et al. 2013). For instance, it has been proposed that the high diversity of marine taxa reached at the beginning of the middle Miocene was a result of temperature increase and favorable oceanographic conditions (Kroh 2007; Schwarzhans 2010). However, these studies predominantly focused on invertebrates, and therefore, understanding of the response of marine communities to the large environmental changes that took place in the last 23 Myr in Europe remains elusive, as it lacks data from vertebrate communities. Despite the large amount of paleontological information that has been accumulated on Neogene chondrichthyans (chimaeras, sharks, rays, skates) from Europe in the last decades (e.g., Marsili 2008; Antunes and Balbino 2010; Bor et al. 2012; Cappetta 2012; Schultz 2013; Marramà et al. 2019), studies of their diversification trajectories during this time of great environmental change are scarce and based on few localities (Kriwet and Klug 2008; Reinecke et al. 2011; Fuchs 2015; Schwab 2015; Villafaña et al. 2020). Here, we synthesize the rich fossil record of Neogene chondrichthyans from Europe in order to reconstruct their diversification trajectories and paleobiogeographic dynamics across different geographic areas from the past to the present. Our results reveal the distinct biogeographic composition of chondrichthyan faunas during the Neogene of Europe and the potential links between diversification trajectories and global and regional climatic changes. As such, this study advances our understanding of the long-term, regional responses of marine communities to major environmental perturbations.

## Materials and Methods

### Data

**Figure 1:**
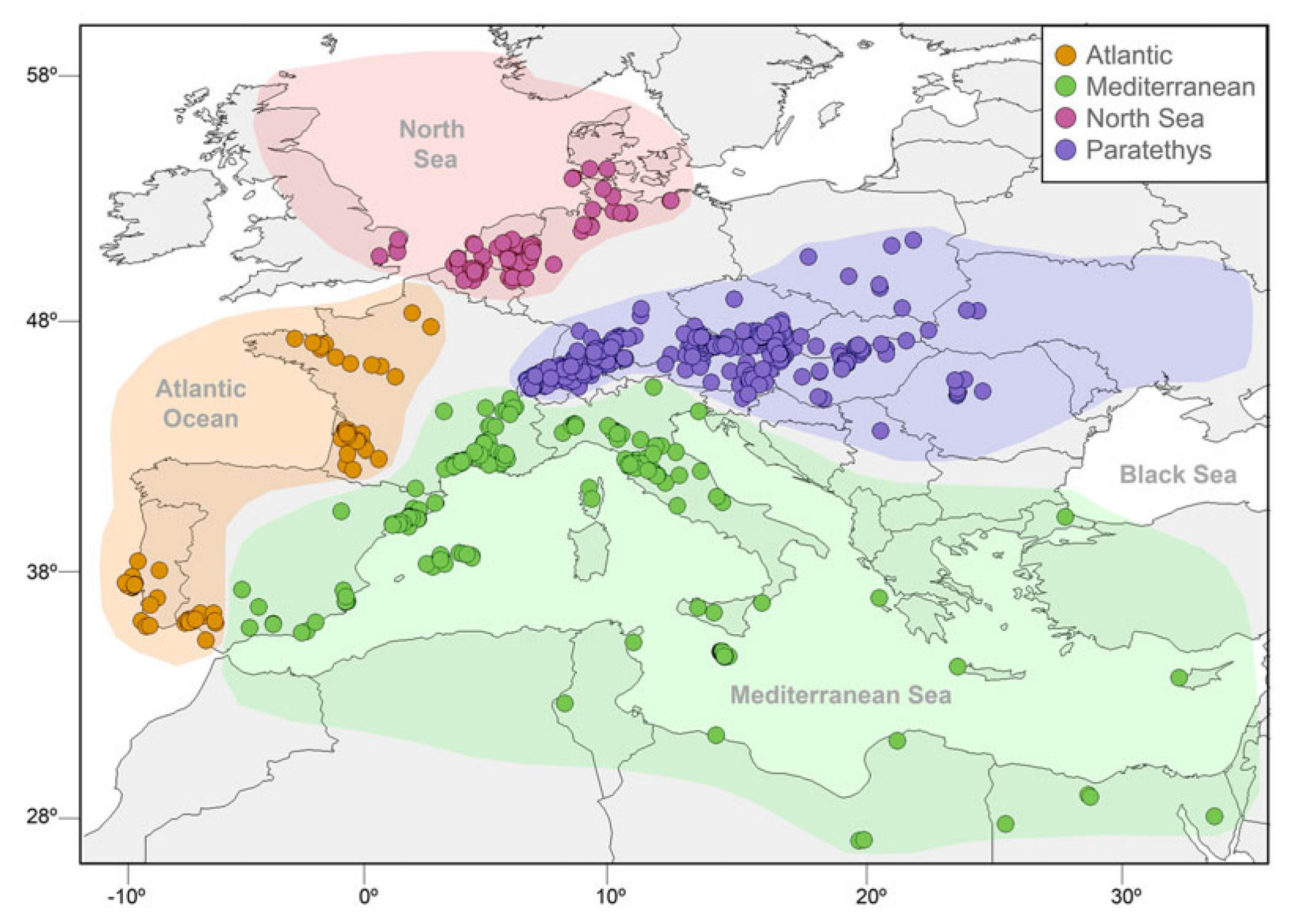
Fossil occurrences of Neogene chondrichthyans from the Atlantic, Mediterranean, North Sea, and Paratethys regions.

We gathered chondrichthyan occurrences at the genus level from the Neogene (23–2.6 Ma) of Europe based on a comprehensive literature quest that consisted of searching for the terms “chondrichthyans”, “fossil”, “Neogene”, and “Europe” in Google Scholar (https://scholar.google.com). This resulted in a list of 122 journal articles, unpublished theses, conference abstracts, and books. This information was complemented with data downloaded from the Paleobiology Database (https://paleobiodb.org) and from museum online collection databases (Supplementary Table S1). Additionally, collections housed in the Natural History Museum of Vienna and the State Museum of Natural History of Stuttgart, Germany, were examined. In total, we collected 4368 occurrences (Fig. 1, Supplementary Table S1), which we assigned to four regions based on the paleogeographic reconstructions proposed by Rögl (1999): Atlantic (n = 433), Mediterranean Sea (*n* = 750), North Sea (*n* = 563), and Paratethys (*n* = 2622). The regional stratigraphic stages (i.e., Paratethys Sea) were updated based on more recent studies (Grunert et al. 2010; Heckeberg et al. 2010; Hohenegger et al. 2014; Kováč et al. 2018). Ambiguous records with unclear taxonomic names (i.e., nonvalid synonyms) or localities (i.e., assigned only to the country level) were excluded from the database. All taxonomic names were updated according to the most recent taxonomic reviews (Cappetta 2012; Pollerspöck and Straube 2021).

It has been demonstrated that deep-water chondrichthyans are differently affected by abiotic factors than shallow-water taxa (e.g., Kriwet and Benton 2004). However, we did not attempt to analyze deep- (<500 m) and shallowwater (>500 m) associations separately, because although several deep-water chondrichthyan faunas have been reported from the Miocene of the Paratethys (e.g., Underwood and Schlögl 2013) and the northern margin of the Tethys (e.g., Cigala-Fulgosi 1996; Adnet 2006), up to now, no deep-water chondrichthyan assemblages from other regions analyzed here have been unambiguously identified. Additionally, dental morphologies of many modern deepwater chondrichthyans are still poorly known, so it is unclear whether taxa (except for most squaliforms) identified in deep-water settings can be associated with such habitat, or if they are representatives of shallow-water taxa but were mixed with deeper-water sediments taphonomically. Consequently, analyzing deep- and shallow-water taxa separately would likely introduce unintended artifacts.

### Analyses

To reconstruct diversification trajectories (i.e., genus richness, origination, and extinction rates), we used the first and last appearance of each genus based on its occurrence, with occurrences distributed in 1 Ma time bins in each region (i.e., Mediterranean, North Sea, and Paratethys). We excluded the Atlantic region from the analysis due to its low number of occurrences (*n* = 433; 10% of the total diversity) and the lack of information on the stratigraphic ages of many localities. Genus richness was calculated per time bin using two approaches: (1) “boundary-crossers” (i.e., number of taxa that cross the boundary of the interval; Foote 2000); and (2) shareholder quorum subsampling (SQS; i.e., fixed coverage of the frequency curve of genus occurrences; Alroy 2010). We used 1000 iterations and quorums of 0.4, 0.6, and 0.8. This last approach was implemented because it accounts for differences in sampling effort, unlike the other approaches, which are prone to sampling biases (Alroy 2010). However, a strong positive correlation between genus richness (estimated using the boundary-crosser method) and the subsampled genus richness (estimated using the SQS method) might suggest that sampling bias is relatively systematic in time (Supplementary Table S2). Origination and extinction rates were estimated as described in the per capita rates of Foote (1999). Origination rates (Eq. 1) are estimated as:

(1) $$p=-\text{ln}\left[N_{bt}/\left(N_{bt}+N_{b}\right)\right]$$

and extinction rates (Eq. 2) as:

(2) $$q=-\text{ln}\left[N_{bt}/\left(N_{bt}+N_{t}\right)\right]$$

where *N*_bt_ is the number of taxa crossing both bottom and top interval boundaries, *N*b is the number of taxa crossing the bottom boundary, and *N*_t_ is number of taxa crossing the top boundary. Singletons were excluded from the origination and extinction rate estimates as proposed by Foote (1999).

To reconstruct the overall extinction magnitude of European chondrichthyans from the Neogene to the Recent, we estimated the Lyellian percentages, that is, the proportion of Neogene genera still living in each region today. Information on present-day distributions of each genus was obtained from the Ocean Biogeographic Information System (2021) and FishBase (Froese and Pauly 2021). Paratethys records were compared against the present-day Mediterranean occurrences. Differences in the proportions of extinct genera across regions were compared using χ^2^ tests. We further made comparisons at higher taxonomic levels (i.e., order and family). We did so by only using the Mediterranean fauna as a comparative region because of its high diversity of chondrichthyans (86 species; Cariani et al. 2017; Ebert and Dando 2020) and because its fossil chondrichthyan record was intensively studied in the past (e.g., Cappetta 2012 and references therein; this study). All analyses were made in R (R Core Team 2021) using the Divdyn library (Kocsis et al. 2019).

## Results

### Taxonomic Composition

The Neogene chondrichthyan fauna comprises 102 genera (Supplementary Table S1) representing four superorders, 12 orders, and 41 families (Table 1). At the superorder level, galeomorphs were the most dominant (i.e., >50%) in the four regions followed by squalomorphs (11–26%). Holocephalans were the least abundant (4%, 5 out of 102), with the Atlantic region displaying the highest number of genera (7%, 4 out of 54). Fossils of this group have not been recorded from the Mediterranean region so far. At the order level, carcharhiniforms and lamniforms were the most abundant groups in the four regions (24–31% and 19–29% of genera, respectively; Table 1, Supplementary Tables S3–S6). Within batoids, the order Myliobati-formes (11–17%) was the best-represented group. At the family level, carchahinids were the most abundant shark group in the Atlantic (11%, 6 out of 54), Mediterranean (10%, 7 out of 70), and Paratethys (10%, 7 out of 69) regions, whereas lamnids (11%, 7 out of 62) were the most common shark family in the North Sea (Table 1). Among batoids, the family Myliobatidae was the most abundant in the Atlantic (7%, 4 out of 54), Mediterranean (6%, 4 out of 70), and Paratethys (6%, 4 of 69) regions, whereas rajids (6%, 4 out of 62) were the most abundant batoids in the North Sea region (Table 1).

### Diversification Trajectories

**Figure 2:**
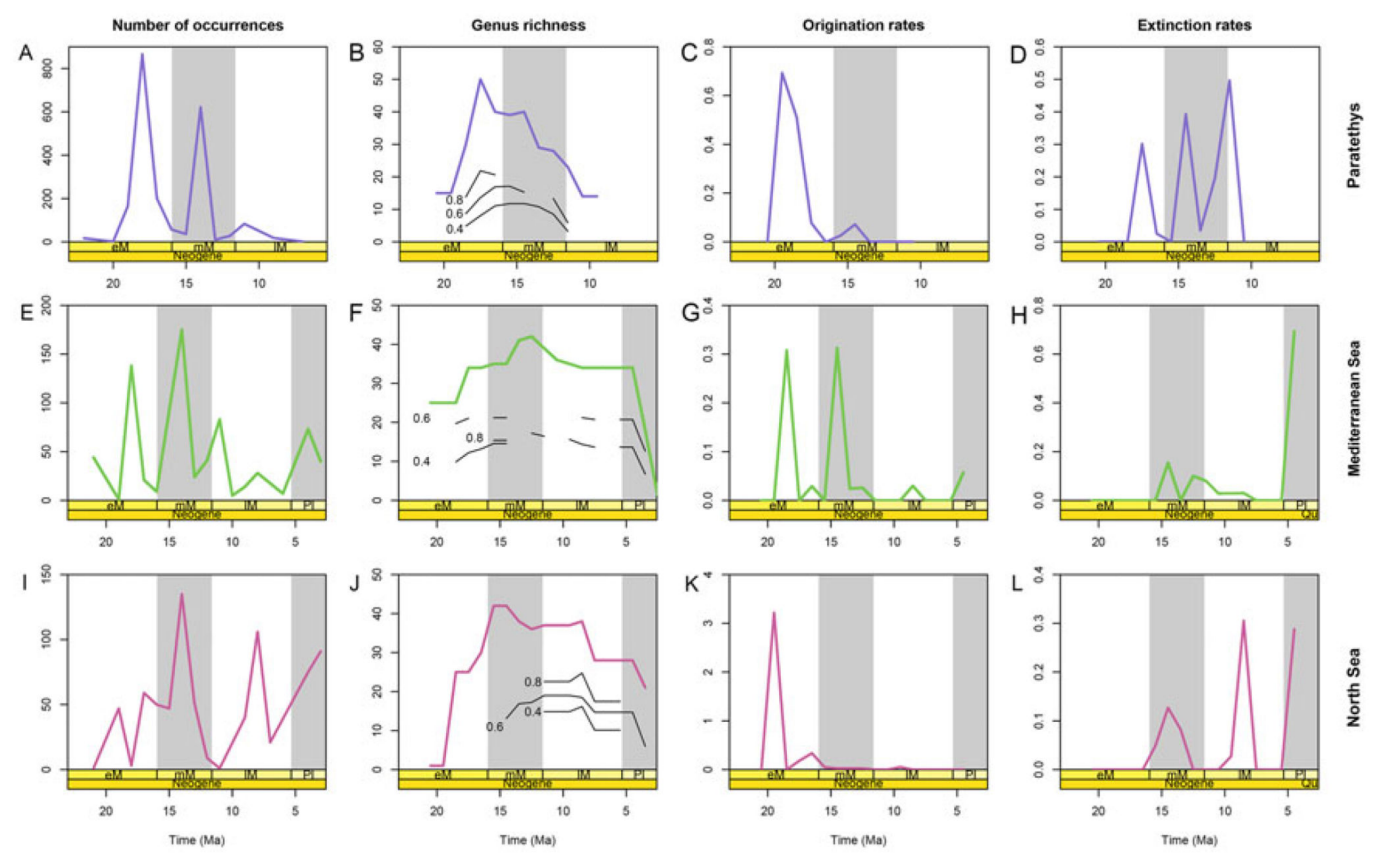
Diversification trajectories of European chondrichthyans. A, E, I, Number of occurrences; B, F, J, standing generic richness; C, G, K, origination rates; and D, H, L, extinction rates. The confidence intervals around the lines were excluded for clarity. Abbreviations: early Miocene (eM), middle Miocene (mM), late Miocene (lM) and Pliocene (Pl). Quorums used to estimate genus richness based on SQS are represented with black lines

The diversity analyses showed marked differences between regions (Fig. 2, Table 2). In the Paratethys, the number of occurrences and genus richness increased and reached maximum values around the early Miocene, but subsequently decreased toward the late Miocene (Fig. 2A,B). In the Mediterranean (Fig. 2E,F) and North Seas (Fig. 2I,J), the number of occurrences and genera increased during the early Miocene, reaching maximum values around the middle Miocene, and then decreasing toward the early Pliocene. The only significant correlation between regions in terms of genus richness was found to be between the Mediterranean and North Sea regions (*r* = 0.80, *p* < 0.05; Table 2).

In terms of origination rates (Fig. 2C,G,K), the Paratethys and the North Sea showed a main pulse during the early Miocene (Fig. 2C, K), whereas in the Mediterranean, two main pulses were observed in the early and middle Miocene (Fig. 2G). There was no significant correlation in origination rates between regions (Table 2). Extinction rates presented substantial pulses during the entire time studied in all regions (Fig. 2D,H,L), with the highest peaks taking place in the Paratethys during the late Miocene (Fig. 2D). In the Mediterranean, there was one main extinction pulse during the Pliocene (Fig. 2H). In the North Sea, there were two main extinction pulses in the late Miocene and Pliocene (Fig. 2L). Extinction rates were significantly correlated between the Mediterranean and North Sea region (*r* = 0.49, *p* < 0.05; Table 2) and the Mediterranean and the Paratethys (*r* = 0.59, *p* < 0.05; Table 2).

The trajectories observed seem to be independent of the method used (Supplementary Table S2). For all regions, the boundary-crosser method was significantly correlated with at least one of the quorums used to estimate the taxonomic richness based on SQS. For instance, in the Paratethys region, the taxonomic richness based on SQS (quota = 0.8) significantly correlates with the boundary-crosser method results (*r* = 0.97, *p* < 0.05) (Supplementary Table S2).

### Biogeography

**Figure 3:**
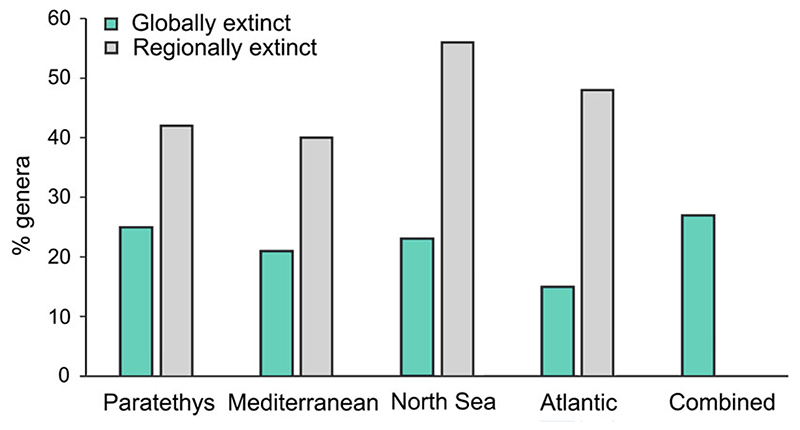
Percentages of extinct/living chondrichthyan genera at each region, at global scale (i.e., the taxon is no longer present in the global ocean) and regional scale (i.e., the taxon was extirpated from the region but is still living elsewhere).

The comparison between the fossil and current distributions (Fig. 3, Supplementary Tables S3–S6) at the genus level showed that 27% (28 out of 102; see “combined” bar in Fig. 3) of genera from the Neogene of Europe are now globally extinct (e.g., *Megascyliorhinus*, *Otodus*, and *Striatolamia*). The proportion of globally extinct genera was only different between the Paratethys (25%, 18 out of 71; Fig. 3) and Atlantic faunas (15%, 8 out of 54; χ^2^ = 3.84, df = 1, *p* = 0.049; Table 3). The proportion of regionally extinct genera was much higher (40–56%; Fig. 3), but there were no differences between regions (*p* >0.05 in all cases; Table 3). The biogeographic distribution of European chondrichthyans among the different regions from the Neogene to the Recent is shown in Supplementary Tables S3–S6.

At the order level, 12 out of 14 extant orders (86%) of chondrichthyans are present in the Neogene of Europe. Using the current diversity of the Mediterranean Sea for comparison, 12 extant orders are shared with the Neogene. Two of the shared orders are absent from the Mediterranean Sea today. As such, orectilobiforms and pristiophoriforms are found in the Neogene, but are absent today in the Mediterranean Sea (Supplementary Table S7). Carcharhiniforms, lamniforms, myliobatiforms, and squaliforms display the highest number of genera both in the Neogene and today (between 11% and 17% of the Neogene generic diversity and between 16% and 18% of today’s diversity in the Mediterranean Sea; Supplementary Table S7). Although chimaeriforms and torpediniforms have not been recovered from the Neogene, they are present today; however, these are the least genus-rich chondrichthyan orders in the Mediterranean Sea today (1% and 2% of the total diversity, respectively; Supplementary Table S7).

At the family level, 40 out of 66 extant families of chondrichthyans were present in the Neogene of Europe (61%). Comparisons based on the Mediterranean Sea show that there are 36 chondrichthyan families shared between the Neogene and today. From these, 27 families (75%) are present today, with 24 (67%) present in both the Neogene and today (Supplementary Table S7). Carcharhinidae was the most genus-rich family in the Neogene, comprising 10% of the diversity (7 out of 70 fossil genera), whereas Rajidae and Dasyatidae are the most diverse families in the Mediterranean Sea today, comprising 11% and 9% of the current diversity (5 and 4 out of 45, respectively; Supplementary Table S7). The family Lamnidae represents one of the second most genus-rich groups in the Neogene (6%, 4 out of 70) and today (7%, 3 out of 45). Fossil specimens of aetobatids, chlamydoselachids, ginglymostomatids, hemigaleids, mitsukurinids, otondontids, plesiobatids, pristiophorids, and rhinids have been recovered from the Neogene, but are absent today in the Mediterranean Sea. Chimaerids, oxynotids, and torpedinids represent the only families not recovered from Neogene localities, but are present today in the Mediterranean region (Supplementary Table S7).

## Discussion

### Diversification Trajectories

**Figure 4:**
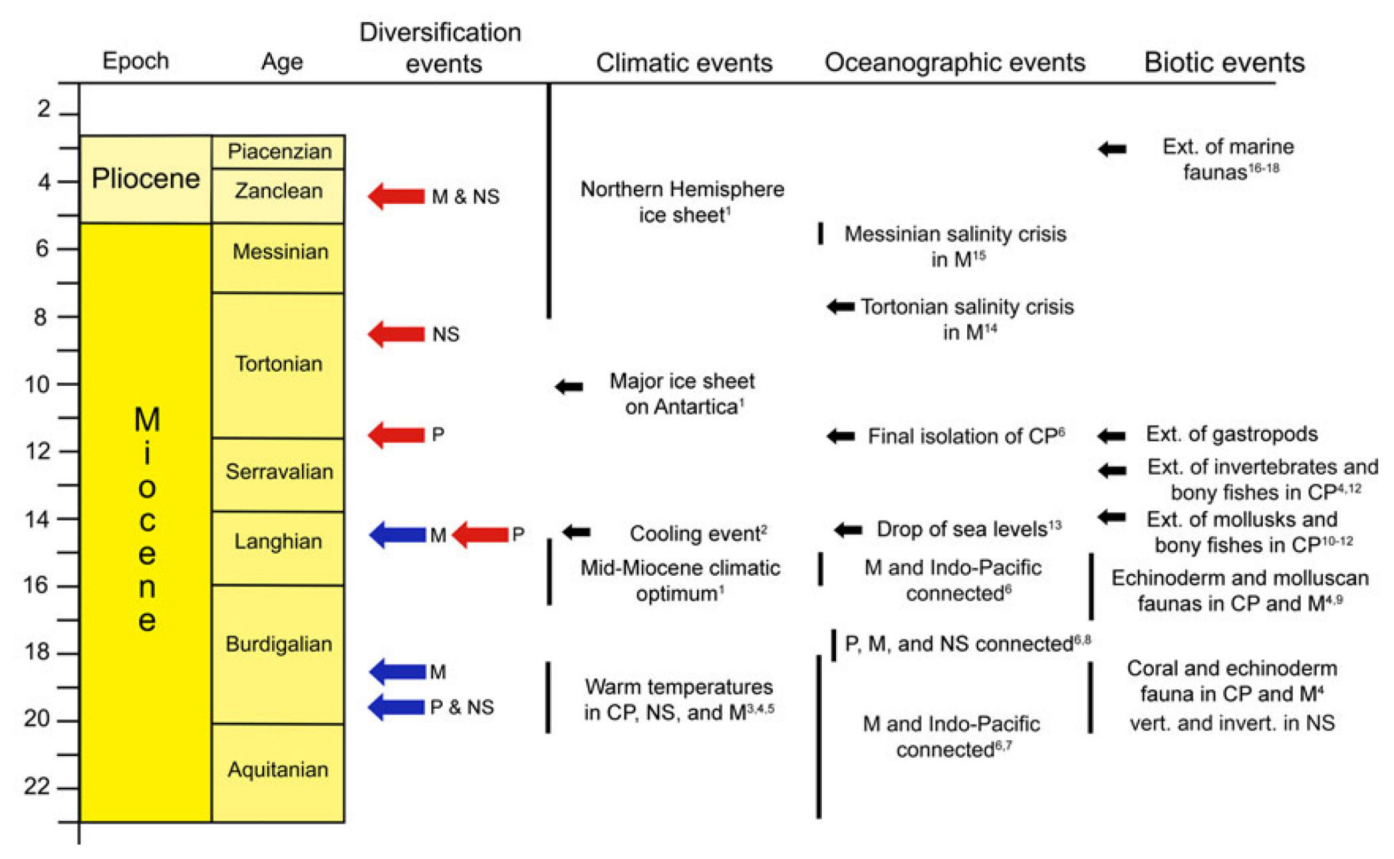
Climatic, oceanographic and biotic events through the Neogene and their relation with the origination (blue arrows) and extinction (red arrows) of Mediterranean (M), North Sea (NS), and Paratethys (P) chondrichthyans. 1: Westerhold et al. (2020); 2: Abreu and Haddad (1998); 3: Nebelsick (1992); 4: Kroh (2007); 5: Gürs and Janssen (2002); 6: Rögl (1999); 7: Harzhauser et al. (2007); 8: Reinecke et al. (2011); 9: Harzhauser et al. (2003); 10: Harzhauser and Piller (2007), 11: Piller et al. (2007); 12: Bannikov et al. (2018); 13: Hohenegger et al. (2014); 14: Krijgsman et al. (2000); 15: Krijgsman et al. (2010); 16: Todd et al. (2002); 17: Rivadeneira and Marquet (2007); 18: Villafaña and Rivadeneira (2014). CP, Central Paratethys; NS, North Sea.

Our analyses revealed marked differences in Neogene chondrichthyan diversification trajectories between the studied regions (Fig. 2; Supplementary Fig. S1), likely reflecting the impact of paleo-environmental conditions that occurred at different spatial scales across Europe and the globe (Fig. 4). For instance, the peak in diversity observed in the Paratethys Sea in the early Miocene (~17.5 Ma), which is evidenced by the high genus richness and origination rates of the time, was coeval with warm temperate conditions during the Eggenburgian (~20.4–18.3 Ma; Nebelsick 1992), which may have also promoted elevated invertebrate origination rates (Kroh 2007). Similarly, in the North Sea, the highest number of occurrences and genera took place in the middle Miocene, while origination rates peaked in the early Miocene (19.5 Ma; Fig. 2). Around this time, specifically between ~19 and 14.5 Ma, the North Sea experienced a warming event due to the incursion of warm-temperate Atlantic waters through a southwest-oriented sea passage (Gürs 2001) that has been associated with increased species richness in vertebrates and invertebrates (e.g., Gürs and Janssen 2002; Kowalewski et al. 2002; Moths et al. 2010; Schwarzhans 2010; Reinecke et al. 2011). In the case of the Mediterranean Sea, there is a steep increase in generic diversity in the early and middle Miocene and two origination peaks at 18.5 and 14.5 Ma. These peaks coincide with the two intervals when the Mediterranean Sea and the Indo-Pacific were connected (23–18 Ma and ~16–15 Ma; Rögl 1999), which resulted in a temperature increase in the region (Harzhauser et al. 2007; Vertino et al. 2014). Taken together, these results suggest that increases in Neogene chondrichthyan diversity coincided with regional warming events during the early and middle Miocene. In current marine systems, species richness tends to be higher in warm areas (e.g., the tropics) than in colder environments (Hillebrand 2004; Kinlock et al. 2018). One of the possible explanations of this temperature dependence for diversity is the kinetic energy or temperature hypothesis, which postulates that high temperatures increase metabolic rates, promoting higher rates of speciation, ultimately leading to greater diversity (Tittensor et al. 2010).

The extinction peaks in the different regions also coincide with regional and global climatic and oceanographic events (Fig. 4). The highest extinction peak in the Paratethys at 11.5 Ma coincides with the isolation of the Central Paratethys Sea from all surrounding marine environments during the Sarmatian/Pannonian (11.6 Ma; Rögl 1999). This isolation caused a gradual change from marine to freshwater conditions, triggering the regional Sarmatian–Pannonian extinction event that resulted in the disappearance of more than 90% of gastropods (Harzhauser and Piller 2007; Borgh et al. 2013). The second-highest extinction peak in the Paratethys (14.5 Ma) coincides with an intense cooling event (14.25 Ma; Abreu and Haddad 1998) that could have been responsible for the extinction of mollusks, bony fishes, and foraminifera in the middle Badenian (Harzhauser and Piller 2007; Piller et al. 2007; Borgh et al. 2013; Hohenegger et al. 2014; Bannikov et al. 2018). In the North Sea region, the first main extinction peak took place at 8.5 Ma, after the connection to the Atlantic Sea was closed (14.5 Ma; Gürs 2001) and a major ice sheet on Antarctica was reestablished (14 Ma; Westerhold et al. 2020). Around 4.5 Ma, there was another extinction peak both in the North Sea and Mediterranean regions, coinciding withthe onset of the Northern Hemisphere glaciation (Westerhold et al. 2020). Our results indicate high extinction rates in European chondrichthyans mostly from the middle Miocene onward that coincided with a global decline in sea temperature linked to the polar glaciation (Westerhold et al. 2020). Indeed, cooling events in Cenozoic have also been linked to the global decline in diversity of lamniform sharks (Condamine et al. 2019). As such, the chondrichthyan extinctions in the Neogene of Europe could be related to cooling events.

Overall, our results indicate that chondrichthyan diversification trajectories during the Neogene of Europe coincided with regional and global warming and cooling events (Fig. 4). In line with this observation, sea temperature has been shown to be a major driver of chondrichthyan species richness today (Tittensor et al. 2010; Guisande et al. 2013). Nevertheless, to be able to postulate a causal link between ocean temperature and peaks in chondrichthyan origination and extinction rates, future research would need to analyze the effect of sea temperature in origination and extinction rates and their interaction with the ecological and life-history traits of each taxon.

### Chondrichthyan Faunas from Europe: Neogene versus Recent

Twenty-seven percent of the genera that inhabited the marine environments of Europe during the Neogene are now globally extinct (Fig. 3), with as much as 56% being regionally extinct (i.e., in the North Sea; Supplementary Table S5). The level of extinction found at the generic level in the Neogene of Europe is much higher than in other areas. For instance, only 3% of the genera from the Neogene of the Pacific of South America (Villafaña and Rivadeneira 2014, 2018) are now globally extinct, and 34% are regionally extinct. Similarly, only 13% of the genera from tropical America (Carrillo-Briceño et al. 2018) are now globally extinct. We attribute these differences in extinction intensities to the large destruction of marine habitats in Europe associated with the drying of the Paratethys and the fluctuation of sea levels in the Mediterranean Sea (Rögl 1999; Brunović et al. 2020). In contrast, despite the significant oceanic changes associated with the rise of the Isthmus of Panama and the closure of the Central American Seaway (Klaus et al. 2011; Montes et al. 2015; O’Dea et al. 2016), the loss of epicontinental seas is not recorded in the Pacific of South America or in tropical America (Miller et al. 2005; Le Roux et al. 2016). Therefore, the intense oceanographic events ultimately resulting in the vanishing of the Paratethys may have had a more significant effect on chondrichthyan faunas in Europe relative to those from the Americas. Indeed, habitat loss is linked to chondrichthyan extinctions in both the fossil record and modern seas (Cione et al. 2007; Pimiento et al. 2017; Dulvy et al. 2021). Nevertheless, to further unveil the mechanisms of chondrichthyan extinctions in Europe, and specifically the role of habitat loss, future studies should analyze how sea-level changes affect species diversification in the region.

As expected, the observed changes in the generic composition vary with taxonomic level. As such, the proportion of genera across orders is similar between the past to the present, with 12 extant orders being found both in the Neogene of Europe and today in the Mediterranean Sea. However, Torpediniformes and Chimaeriformes are absent from the Neogene and Orectolobiformes and Pristiophoriformes are found in the Neogene but are absent today in the Mediterranean Sea (Supplementary Table S7). Indeed, it has been shown that the chondrichthyan fossil record is largely conserved at the order level relative to today (Pimiento and Benton 2020; Paillard et al. 2021). At the family level there are larger differences, with nine families (Supplementary Table S7) present in the Neogene of the Mediterranean Sea but absent today, and three families (Chimaeridae, Oxynotidae and Torpedinidae) found in the Mediterranean Sea today, but absent from the Neogene. Our results therefore suggest that Neogene chondrichthyan faunas from Europe suffered a significant taxonomic loss relative to other regions, as well as a possible rearrangement of taxonomic composition at the order and family levels.

## Conclusions and Perspectives

Our analysis provides first steps toward synthesizing and understanding the macroevolutionary diversification trajectories and paleobiogeographic changes of chondrichthyans during the Neogene in Europe. Increases in number of genera and peaks of origination seem to be related to warming events, whereas the highest extinction peaks appear to be related to cooling events. The biogeographic comparison between past and present shows that global and regional extinctions of chondrichthyans in Europe were much higher than in other regions. The present study nevertheless should be considered an initial step toward understanding Neogene diversity patterns of Europe; additional paleontological studies are needed to provide additional data for further refinements of the present analyses, including (1) more precise dating of fossil inventories; (2) more specific information related to the collection method used; (3) inclusion of ecological and life-history traits of each taxon in order to understand the biogeographic dynamics through the time; (4) establishment of the correct ecology for extinct taxa based on detailed comparisons with living taxa to better distinguish between deep- (<500) and shallow-water (>500 m) chondrichthyan taxa to determine the influence of abiotic factors on their diversity fluctuations and extinction risk; and (5) more robust tests of the role of environmental drivers based on new paleoceanographic reconstructions.

## Supplementary Material

Table S1

Table S2

Table S3

Table S4

Table S5

Table S6

Table S7

## Figures and Tables

**Table 1:** Faunal composition of Neogene European chondrichthyans at superorder, order, and family levels.

	Atlantic		Mediterranean		North Sea		Paratethys	
	*n*	%	*n*	%	*n*	%	*n*	%
A. Superorder								
Galeomorphii	28	52	35	50	34	55	36	51
Squalomorphii	6	11	17	24	9	15	18	26
Batomorphii	16	30	18	26	17	27	15	21
Holocephali	4	7	0	0	2	3	1	1
B. Order								
Carcharhiniformes	17	31	17	24	15	24	20	29
Chimaeriformes	4	7	0	0	2	3	1	1
Hexanchiformes	2	4	5	7	2	3	4	6
Lamniformes	10	19	16	23	18	29	15	21
Myliobatiformes	9	17	11	16	9	15	8	11
Orectoboliformes	1	2	2	3	1	2	1	1
Pristiophoriformes	1	2	1	1	1	2	1	1
Rajiformes	2	4	2	3	5	8	3	4
Rhinopristiformes	4	7	4	6	2	3	3	4
Squaliformes	2	4	11	16	5	8	12	17
Squatiniformes	1	2	1	1	1	2	1	1
Torpediniformes	1	2	0	0	1	2	1	1
C. Family								
Aetobatidae	1	2	1	1	1	2	1	1
Alopiidae	1	2	2	3	1	2	1	1
Arhynchobatidae	0	0	0	0	1	2	0	0
Callorhinchidae	2	4	0	0	2	3	1	1
Carcharhinidae	6	11	7	10	5	8	7	10
Centrophoridae	0	0	2	3	0	0	2	3
Cetorhinidae	0	0	2	3	2	3	2	3
Chimaeridae	1	2	0	0	0	0	0	0
Chlamydoselachidae	0	0	1	1	0	0	1	1
Dalatiidae	1	2	4	6	1	2	4	6
Dasyatidae	3	6	3	4	2	3	2	3
Echinorhinidae	0	0	1	1	1	2	1	1
Etmopteridae	0	0	1	1	0	0	2	3
Ginglymostomatidae	1	2	1	1	1	2	1	1
Gymnuridae	1	2	1	1	1	2	1	1
Hemigaleidae	4	7	3	4	3	5	3	4
Hexanchidae	2	4	4	6	2	3	3	4
Lamnidae	3	6	4	6	7	11	4	6
Megachasmidae	0	0	0	0	1	2	0	0
Mitsukurinidae	0	0	3	4	1	2	2	3
Mobulidae	0	0	1	1	1	2	0	0
Myliobatidae	4	7	4	6	3	5	4	6
Odontaspididae	3	6	3	4	4	6	3	4
Otodontidae	2	4	2	3	2	3	2	3
Oxynotidae	0	0	0	0	1	2	0	0
Plesiobatididae	0	0	1	1	1	2	0	0
Pristidae	2	4	2	3	0	0	1	1
Pristiophoridae	1	2	1	1	1	2	1	1
Pseudocarchariidae	1	2	0	0	0	0	1	1
Rajidae	2	4	2	3	4	6	2	3
Rhincodontidae	0	0	1	1	0	0	0	0
Rhinidae	1	2	1	1	1	2	1	1
Rhinobatidae	1	2	1	1	1	2	1	1
Rhinochimaeridae	1	2	0	0	0	0	0	0
Scyliorhinidae	3	6	3	4	3	5	7	10
Somniosidae	0	0	2	3	0	0	2	3
Sphyrnidae	1	2	1	1	1	2	1	1
Squalidae	1	2	1	1	2	3	1	1
Squatinidae	1	2	1	1	1	2	1	1
Torpedinidae	1	2	0	0	1	2	1	1
Triakidae	3	6	3	4	3	5	2	3

**Table 2:** Spearman moment-product correlation for diversity trajectories between paired regions.^a^

Variable	Mediterranean-North Sea	Mediterranean-Paratethys	Paratethys-North Sea
Genus richness	**0.80**	−0.11	0.14
Origination rate	−0.06	0.07	0.34
Extinction rate	**0.49**	**0.59**	0.22

a Significant values (*p* < 0.05) are in bold.

**Table 3:** Differences in the proportions of extinct genera at global and regional scales between regions using χ^2^ test.^a^

Scale	Region	Atlantic	Mediterranean	North Sea	Paratethys
Global	Atlantic	—	0.144	0.200	**0.049**
	Mediterranean	0.144	—	0.852	0.601
	North Sea	0.200	0.852	—	0.479
	Paratethys	**0.049**	0.601	0.479	—
Regional	Atlantic	—	1	0.475	1
	Mediterranean	1.000	—	0.475	1.000
	North Sea	0.475	0.475	—	—
	Paratethys	1	1	0.475	1

a Significant values (*p* < 0.05) are in bold.

## Data Availability

Data available from the Dryad Digital Repository: https://doi.org/10.5061/dryad.34tmpg4pd.
