# Supplementary material for: Diversification trajectories and paleobiogeography of Neogene chondrichthyans from Europe
Source: Paleobiology. Author manuscript; Available in PMC 2023 Aug 10. (PMC7614935; doi:10.1017/pab.2022.40)
Supplement: Table S2 [file EMS183745-supplement-Table_S2.docx]

| Region | SQS 0.4 – BC | SQS 0.6 – BC | SQS 0.8 – BC |
| --- | --- | --- | --- |
| Paratethys | 0.48 | 0.63 | **0.97** |
| Mediterranean | **0.96** | **0.89** | 0.27 |
| North Sea | **0.57** | 0.31 | 0.31 |
