# Supplementary material for: Diversification trajectories and paleobiogeography of Neogene chondrichthyans from Europe
Source: Paleobiology. Author manuscript; Available in PMC 2023 Aug 10. (PMC7614935; doi:10.1017/pab.2022.40)
Supplement: Table S7 [file EMS183745-supplement-Table_S7.docx]

|  |  | Past | | Today | |
| --- | --- | --- | --- | --- | --- |
|  |  | n | % | n | % |
| A) Order | Carcharhiniformes | 17 | 24 | 7 | 16 |
|  | Hexanchiformes | 5 | 7 | 2 | 4 |
|  | Lamniformes | 16 | 23 | 7 | 16 |
|  | Myliobatiformes | 11 | 16 | 8 | 18 |
|  | Torpediniformes | 0 | 0 | 2 | 4 |
|  | Chimaeriformes | 0 | 0 | 1 | 2 |
|  | Orectolobiformes | 2 | 3 | 0 | 0 |
|  | Pristiophoriformes | 1 | 1 | 0 | 0 |
|  | Rajiformes | 2 | 3 | 5 | 11 |
|  | Rhinopristiformes | 4 | 6 | 4 | 9 |
|  | Squaliformes | 11 | 16 | 8 | 18 |
|  | Squatiniformes | 1 | 1 | 1 | 2 |
| B) Family | Aetobatidae | 1 | 1 | 0 | 0 |
|  | Alopiidae | 2 | 3 | 1 | 2 |
|  | Carcharhinidae | 7 | 10 | 2 | 4 |
|  | Centrophoridae | 2 | 3 | 1 | 2 |
|  | Cetorhinidae | 2 | 3 | 1 | 2 |
|  | Chlamydoselachidae | 1 | 1 | 0 | 0 |
|  | Chimaeridae | 0 | 0 | 1 | 2 |
|  | Dalatiidae | 4 | 6 | 1 | 2 |
|  | Dasyatidae | 3 | 4 | 4 | 9 |
|  | Echinorhinidae | 1 | 1 | 1 | 2 |
|  | Etmopteridae | 1 | 1 | 1 | 2 |
|  | Ginglymostomatidae | 1 | 1 | 0 | 0 |
|  | Gymnuridae | 1 | 1 | 1 | 2 |
|  | Hemigaleidae | 3 | 4 | 0 | 0 |
|  | Hexanchidae | 4 | 6 | 2 | 4 |
|  | Lamnidae | 4 | 6 | 3 | 7 |
|  | Mitsukurinidae | 3 | 4 | 0 | 0 |
|  | Mobulidae | 1 | 1 | 1 | 2 |
|  | Myliobatidae | 4 | 5 | 2 | 4 |
|  | Odontaspididae | 3 | 4 | 2 | 4 |
|  | Oxynotidae | 0 | 0 | 1 | 2 |
|  | Otodontidae | 2 | 3 | 0 | 0 |
|  | Plesiobatididae | 1 | 1 | 0 | 0 |
|  | Pristidae | 2 | 3 | 1 | 2 |
|  | Pristiophoridae | 1 | 1 | 0 | 0 |
|  | Rajidae | 2 | 3 | 5 | 11 |
|  | Rhincodontidae | 1 | 1 | 2 | 4 |
|  | Rhinidae | 1 | 1 | 0 | 0 |
|  | Rhinobatidae | 1 | 1 | 1 | 2 |
|  | Scyliorhinidae | 3 | 4 | 2 | 4 |
|  | Somniosidae | 2 | 3 | 2 | 4 |
|  | Sphyrnidae | 1 | 1 | 1 | 2 |
|  | Squalidae | 1 | 1 | 1 | 2 |
|  | Squatinidae | 1 | 1 | 1 | 2 |
|  | Torpedinidae | 0 | 0 | 2 | 4 |
|  | Triakidae | 3 | 4 | 2 | 4 |
